# Differences in Humoral Immune Response against the Type 2 Porcine Reproductive and Respiratory Syndrome Virus via Different Immune Pathways

**DOI:** 10.3390/v14071435

**Published:** 2022-06-29

**Authors:** Wen Li, Yangyang Sun, Shijie Zhao, Zhiying Cui, Yu Chen, Pengli Xu, Jing Chen, Yina Zhang, Pingan Xia

**Affiliations:** 1 College of Veterinary Medicine, Henan Agricultural University, Zhengdong New District Longzi Lake 15#, Zhengzhou 450046, China; lw839466399@163.com (W.L.); syy163yx@126.com (Y.S.); zsj1002935527@163.com (S.Z.); czy11023@163.com (Z.C.); 15290876663@163.com (Y.C.); xpl129012@163.com (P.X.); xpa88@163.com (P.X.); 2 College of Life Science, Henan Agricultural University, Jinshui District, Zhengzhou 450002, China

**Keywords:** PRRSV-2, mucosal immunity, viremia, IgG, SIgA, NAs, in vivo

## Abstract

The intramuscular vaccine is the principal strategy to protect pigs from porcine reproductive and respiratory syndrome virus (PRRSV), However, it is still difficult to control PRRSV effectively. This study infected piglets with PRRSV through intramuscular and intranasal inoculation. Subsequently, viral loads, anti-PRRSV antibody levels, and neutralizing antibodies (NAs) titers in both serum and saliva were monitored for 43 days. Meanwhile, tissues were obtained through necropsy at 43 days post-inoculation (dpi) to detect viral loads. The results indicated that viremia lasted from 3 to 31 dpi in both the inoculation groups, but the viruses survived in the lungs and lymph nodes after viremia clearance. The antibody response was detected from 11 dpi, but the response of NAs was delayed until 3–4 weeks. Furthermore, intranasal inoculation induced lower viral load levels than injection inoculation. In addition, positive SIgA and NAs levels were produced early, with higher levels through intranasal inoculation. Therefore, our data indicated that a more robust antibody response and lower virus loads could be induced by intranasal inoculation, and mucosal inoculation could be a suitable pathway for PRRSV vaccines.

## 1. Introduction

Porcine reproductive and respiratory syndrome (PRRS) is a viral disease caused by the porcine reproductive and respiratory syndrome virus (PRRSV), showing severe reproductive disorders in sows and respiratory symptoms in piglets. Porcine reproductive and respiratory syndrome has profoundly affected the swine industry and caused significant economic losses in the global swine industry in the past 30 years. There are two species, PRRSV-1 (type 1) and PRRSV-2 (type 2), sharing approximately 60% nucleotide sequence identity, and are recently classified as Betaarterivirus suid 1 and Betaarterivirus suid 2, respectively, from the genus Betaarterivirus (EC 52, Online meeting, October 2020) (https://talk.ictvonline.org/taxonomy/p/taxonomy-history?taxnode_id=20171832, accessed on 10 March 2022) [1]. Moreover, there are considerable genetic and virulence differences between the PRRSV species. Since a PRRSV-2 was firstly reported in China in 1996, it has been a significant epidemic strain in China for nearly 30 years. The study indicated that PRRSV-2 modified live virus (MLV) could provide partial heterologous cross-protection against the PRRSV-1 virus, but the PRRSV-1 MLV was ineffective against PRRSV-2 [2,3]. Even though scientists understand PRRSV in virology, evolution, and increasing host immune response, current strategies to control PRRSV are still largely inadequate due to the emerging new PRRSV variants. Various anti-PRRSV strategies have been used to manage the PRRSV infection, such as herd depopulation and repopulation, removal of the PRRSV-positive animals, and vaccination [4,5,6]. However, controlling and eliminating PRRSV in the field is still tricky. Tremendous efforts have been made, such as developing inactivated vaccines, improved live vaccines, recombinant protein vaccines, and DNA vaccines to protect pigs from this economically devastating disease. Among them, only cell-cultured attenuated vaccines could provide limited protection but are challenging to avoid PRRSV persistent infection. Meanwhile, practical experience has revealed that numerous safety and efficacy issues with currently licensed vaccines, including the shedding of modified live virus, reversion to virulence, recombination between field and vaccine strains, and failure to elicit protective immunity against the heterogeneous virus [7]. Moreover, antibody-dependent enhancement (ADE) complicates the pathogenic mechanism of PRRSV to a certain extent. Therefore, the efficacy and safety of attenuated vaccines are a concern for researchers and practitioners. 

The humoral immune response effect on PRRSV is complicated. The PRRSV infection stimulates an antibody response by 7–9 days post-inoculation (dpi) without any evidence of protection against PRRSV infection. Neutralizing antibodies (NAs) play a crucial role in antibody response due to blocking viral infection. The IgG is mainly present in the serum and contributes to limiting viremia. Previous studies established that IgG NAs play a significant role in protecting pigs against PRRSV infection. Serum NAs appear late, typically ≥28 dpi [8]. The delayed response of NAs led to the low efficacy of PRRSV vaccines [4]. Therefore, improving the response of the PRRS vaccine is a crucial factor in enhancing the immune protection of the vaccine. Mucosal vaccination is ideal for controlling infectious diseases inducing systemic and mucosal antigen-specific immune responses. The mucosal immune system is the largest component of the immune system in terms of immune cells deployment and immunoglobulins production. It has evolved to protect the main infectious site: the mucosae.

Most mucosal vaccines are administered via oral and nasal routes. Mucosal vaccines have been successful in the veterinary field, with spraying and drinking water routinely used for mass vaccination in poultry farming. Nasal vaccination can mimic the natural infection of pathogens, the antigens reaching the respiratory cavity and through the mucus layer to elicit specific mucosal and systemic immune responses. The IgA primarily contributes to mucosal system response. Studies on IgA have revealed that secretory IgA antibodies (multimers) had high neutralizing activity against viruses, possibly due to increased cohesion against viral antigens [9]. In recent years, many studies have proved that mucosal immunity is effective in the preventing and controlling some diseases [10]. The mucosal vaccines against the influenza virus had been commercial and used to prevent seasonal influenza [11]. Intranasal vaccination with a lentiviral vector protects against SARS-CoV-2 in preclinical animal models and elicits an immune response in the respiratory tract through an intranasal boost. It results in a >3 log_10_ decrease in viral loads in the lung and reduces local inflammation [12]. As a significant respiratory disease of piglets, there are only a few studies regarding the function of mucosal immunity and SIgA in PRRSV vaccines. Therefore, understanding the mucosal immunity of PRRSV is significant for improving the immune strategy against the disease. 

The PRRSV infection usually induces an inadequate or absent host immune response by inhibiting IFN-α production [13,14], while HeN-3 exhibited different responses. Our previous studies have revealed that the field virus HeN-3 caused a high IFN-α production in PAMs during early infection and slightly inhibited IFN-α production in PAMs during late infection [15]. Moreover, HeN-3 can induce the production of IFN-α in piglets in the first 15 dpi [16]. The HeN-3 infection may cause an IFN-α antiviral response. Based on this difference, we speculated that the HeN-3 might also induce a stronger humoral immune response, and it has the potential to be an MLV candidate strain. In the current study, the primary purpose was to investigate the effects of intranasal and intramuscular inoculation on the humoral immune response, induced by HeN-3 (a PRRSV-2 strain), by detecting viremia, anti-PRRSV antibodies, and NAs in piglets. We had the following two objectives: (1) to monitor the dynamics of viremia and antibodies in a time-dependent manner after inoculation, and (2) to analyze the differences in humoral immune responses after different inoculation strategies. The results confirmed that a more robust antibody response and lower virus loads could be induced using intranasal inoculation. Thus, mucosal vaccination could be a suitable pathway for PRRSV vaccines.

## 2. Materials and Methods

### 2.1. Cells and Virus Strain

Porcine alveolar macrophages (PAMs) for neutralizing tests were isolated from six-weeks-old PRRSV-negative healthy pigs through bronchoalveolar lavage after necropsy. The PRRSV-2 strain HeN-3 (GenBank ID: ON645930) was stored in our laboratory, as previously described [15].

### 2.2. Animal Experiments

The infection experiments were undertaken at the College of Veterinary Medicine at Henan Agricultural University, Zhengzhou, China. We obtained twelve 20-days-old healthy piglets from a conventional PRRSV-free farm with similar genetic backgrounds. They were free from PRRSV, porcine circovirus-2, pseudorabies virus, and swine influenza virus by analyzing through real-time quantitative PCR (RT-qPCR). Piglets were randomly divided into three groups of four animals (intranasal, injection, and control) and housed in three separate rooms. Piglets in test groups were inoculated with 10^6^ TCID_50_ PRRSV HeN-3 through intranasal and intramuscular inoculation (in nape) respectively, and piglets in the control group were inoculated with equal volumes of the cell culture medium. Serum and saliva in all the groups were respectively collected for viral loads and antibody titers at 0, 3, 7, 11, 15, 19, 23, 27, 31, 35, 39, and 43 dpi. At 43 dpi, the PAMs, submandibular lymph nodes, and inguinal lymph nodes of piglets were obtained for viral loads detection after necropsy under clean conditions. 

### 2.3. Sample Treatment

#### 2.3.1. Serum Samples

Whole blood was collected from the anterior vena cava of each piglet. The serum was obtained by centrifuging at 3000 rpm for 10 min, and stored at −80 °C. 

#### 2.3.2. Saliva Samples

Several sterile swabs were placed inside the mouth of the respective pig, and the saliva was allowed to soak within the swabs. The swabs were centrifuged for 10 min at 3000 rpm in 50 mL filtered tubes and stored at −80 °C. 

#### 2.3.3. Tissue Samples

Tissue samples, such as lungs, submandibular lymph nodes, and inguinal lymph nodes were obtained under clean conditions. All the tissue samples were stored at −80 °C.

#### 2.3.4. PAMs Samples

The PAMs samples were collected and washed with phosphate-buffered saline (PBS) containing 1× penicillin-streptomycin liquid (Solarbio, Beijing, China) by bronchoalveolar lavage in a sterile room and suspended in the Roswell Park Memorial Institute (RPMI) 1640 medium (Thermo Fisher Scientific, Waltham, MA, USA) after centrifugation. Then, the PAMs were seeded in RPMI-1640 medium supplemented with 10% Fetal Bovine Serum (FBS) (Thermo Fisher Scientific, Waltham, MA, USA) and 1× penicillin-streptomycin liquid at a 1 × 10^6^ cells/well ratio and incubated at 37 °C and 5% CO_2_.

### 2.4. PRRSV Viral Loads Detection

As previously described, the extraction of the total RNA of the virus was performed by using TRIzol based on the manufacturer’s instructions (Vazyme, Nanjing, China). Then, RNA was reverse transcribed into cDNA by using the HiScript II 1st Strand cDNA Synthesis Kit (Vazyme, Nanjing, China). The generated cDNA was amplified using quantitative PCR with the following specific primers (5′-AAACCAGTCCAGAGGCAAGG-3′/5′-GCAAACTAAACTCCACAGTGTAA-3′). The RT-qPCR was developed to detect PRRSV using ChamQ Universal SYBR qPCR Master Mix (Vazyme, Nanjing, China). The RT-qPCR reaction was performed using the CFX 96 Touch System (Bio-Rad, Hercules, CA, USA) in a 96-well plate. The cycling conditions were denaturation for 10 min at 95 °C, amplification for 40 cycles, denaturation for 5 s at 95 °C, annealing, and extension for 34 s at 60 °C. A standard curve was plotted using the plasmid standards. The melt-curve analysis of the RT-qPCR products was performed according to the manufacturer’s protocol to confirm the specific amplification. The viral mRNA copies were determined using the standard curve generated from specific PCR products as the template [17].

### 2.5. Anti-PRRSV Antibodies Detection

The anti-PRRSV serum IgG and saliva SIgA were captured using a modified enzyme-linked immunosorbent assay (ELISA) [18]. The PRRSV HeN-3 was purified using density gradient ultracentrifugation and diluted to 10 µg/mL in coating buffer and poured into 96-well ELISA plates (Corning, NY, USA) overnight at 4 °C for serum anti-PRRSV IgG measurement. After washing three times with PBS containing 0.05% Tween-20 (PBST), the plates were blocked using PBST containing 1% BSA (Solarbio, Beijing, China) (blocking buffer) at 37 °C for 2 h and then washed three times with PBST. Then, the serum samples were 40-fold diluted in blocking buffer and incubated at 37 °C for 1 h. After washing five times, HRP-labeled rabbit-anti-porcine IgG (Abcam, Cambridge, MA, USA) was diluted using the blocking buffer with a 1:5000 ratio and incubated at 37 °C for 1 h. After washing five times, TMB substrate (Solarbio, Beijing, China) was added to the plates and incubated at 37 °C for 15 min, and the reaction was stopped by adding 2 M H_2_SO_4_. The absorbance was measured at 450 nm through a microplate reader. For saliva anti-PRRSV SIgA measurement, the saliva samples were two-fold diluted, and secondary antibody HRP-labeled goat-anti-porcine IgA (Abcam, Cambridge, MA, USA) was used. The remaining steps were the same as above. The presence or absence of the antibody was determined by evaluating the sample/positive (S/P) ratio, where S/P = (sample OD_450_−negative-control OD_450_)/(positive-control OD_450_−negative-control OD_450_). An S/P ratio of 0.4 or greater was determined to be positive.

### 2.6. Anti-PRRSV NAs Detection

Anti-PRRSV NAs were detected by a virus-neutralizing (VN) test based on PAMs as described previously [19]. A total of 1 × 10^6^ PAMs were seeded within each well of a 24-well-plate (Corning, NY, USA) and cultured at 37 °C overnight. Serum and saliva samples were inactivated for 30 min at 56 °C and then subjected to two-fold dilutions from 1:2 to 1:32 in the RPMI-1640 medium. Later, the diluted samples (100 μL per well) were incubated with an equal volume of PRRSV (100 TCID_50_) at 37 °C for 1 h to form complexes. The samples collected at 0 dpi were used as the negative control. The complexes were added to PAMs. Two hours later, the medium was replaced with a fresh RPMI-1640 medium with 10% FBS and incubated at 37 °C for 48 h. Total RNAs were extracted from cell cultures, and RT-qPCR quantified the viral loads. The reciprocal value of the highest serum or saliva dilution causing a 70% reduction of virus number was defined as the virus NAs titer.

### 2.7. Statistical Analysis

The statistical analyses were performed using GraphPad Prism 8.0 software (GraphPad Software Incorporated, San Diego, CA, USA). The statistical difference was calculated using the *t*-test and reported as follows, *, *p* < 0.05; **, *p* < 0.01; ***, *p* < 0.001; ****, *p* < 0.0001.

## 3. Results

### 3.1. Viremia Detection in Serum Samples

**Figure 1:**
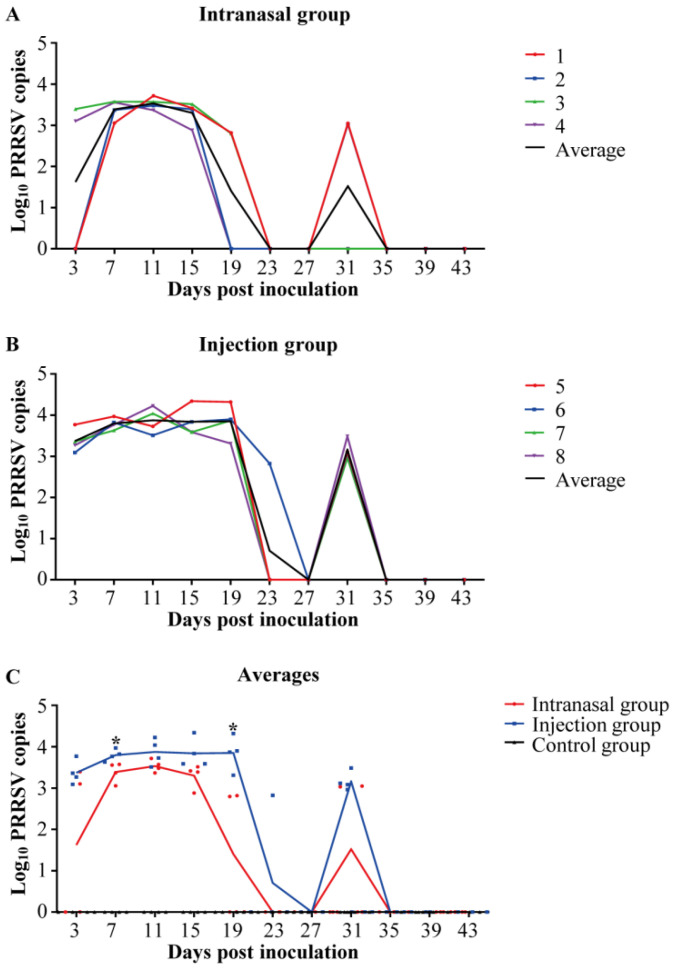
Viremia of PRRSV-inoculated piglets. Viral loads were quantified in serum through RT-qPCR in piglets to assess the viremia of PRRSV. Twelve 20-days-old healthy piglets were divided into three groups (*n* = 4 in each group). Piglets in the test groups were inoculated with attenuated PRRSV (10^6^ TCID_50_) through intranasal and intramuscular inoculation respectively, and piglets in the control group were inoculated with equal volumes of the cell culture medium. Serum samples were collected at 0, 3, 7, 11, 15, 19, 23, 27, 31, 35, 39 and 43 dpi. Piglets in the control group were PRRSV-negative throughout the study. A and B: Colored lines represent individual animals, and black lines represent the mean value for each group. Numbers (1–8) represent the individual pig numbers. (**A**) Viremia of piglets in the intranasal group. (**B**) Viremia of piglets in the injection group. (**C**) The averages of viremia in each group. Statistical differences were determined using the *t*-test (* *p* < 0.05). Comparisons were undergone between the Intranasal and the Injection groups.

Viral loads were quantified in the serum of piglets through RT-qPCR to assess the viremia of PRRSV. Piglets in the control group stayed PRRSV-negative throughout the study. Figure 1A, B show that, most PRRSV-inoculated piglets developed viremia at 3 dpi and peaked at 11 dpi. They were symptom-free, although their viremia lasted until 31 dpi. However, intranasal inoculation induced lower viremia levels than injection inoculation (Figure 1C). 

### 3.2. PRRSV Viral Loads Detection in Tissue Samples

**Figure 2:**
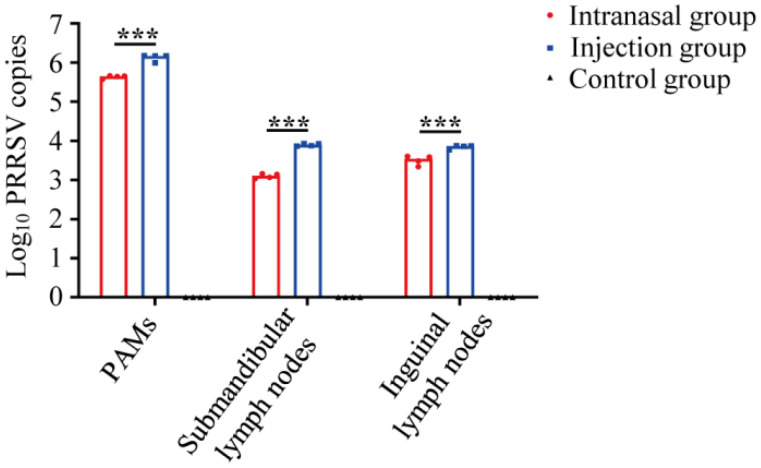
Viral loads of tissues after inoculation. Viral loads were quantified in tissues through RT-qPCR in piglets. Tissues were obtained after necropsy at 43 dpi. Piglets in the control group were PRRSV-negative. Statistical differences were determined using the *t*-test (*** *p* < 0.001). Comparisons were between the Intranasal and the Injection groups.

The RT-qPCR detected the PRRSV genomes in all the tissue samples. Lungs, submandibular lymph nodes, and inguinal lymph nodes from piglets in all three groups were obtained after necropsy at 43 dpi. Moreover, PAMs were collected in a sterile room from the collected lungs. The PAMs, submandibular lymph nodes, and inguinal lymph nodes from the inoculated groups were PRRSV-positive. All samples from the control group were PRRSV-negative. As shown in Figure 2, viral loads of the injection group were significantly higher in all tissues than that of the intranasal group, indicating that intranasal inoculation causes in fewer viral loads in tissues, and the virus could survive in some tissues even after initial clearance in serum. 

### 3.3. Anti-PRRSV IgG Detection in Serum Samples

**Figure 3:**
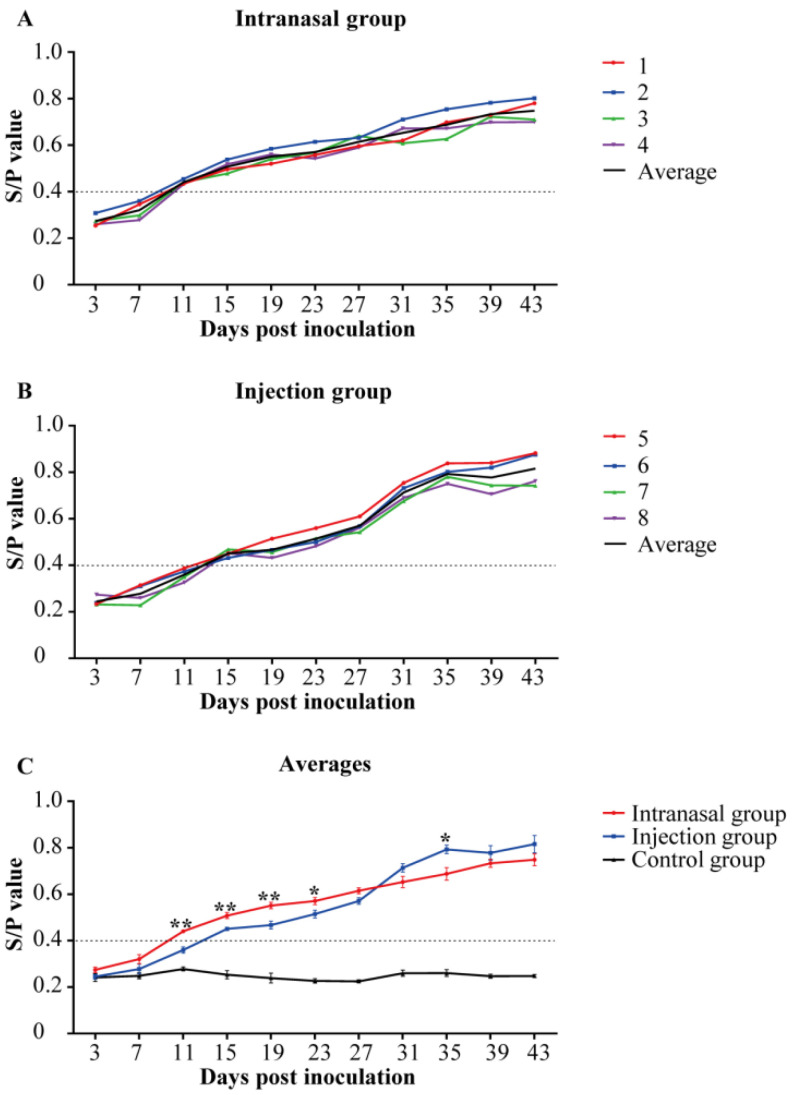
Anti-PRRSVserum IgG levels. The anti-PRRSV serum IgG levels were quantified using ELISA in piglets (*n* = 4 in each group) to assess the anti-PRRSV antibody response. Serum samples collected at 0 dpi were served as the negative control. The cut-off for positivity was 0.4. Piglets in the control group were IgG-negative throughout the study. (**A**,**B**): Colored lines represent the individual animals, and black lines represent the mean value of each group. Numbers (1–9) represent the individual pig numbers. (**A**) Anti-PRRSV serum IgG levels in the intranasal group. (**B**) Anti-PRRSV serum IgG levels in the injection group. (**C**) The averages of serum IgG levels within each group. The graph illustrates the mean ± SEM. Statistical differences were determined using the *t*-test (* *p* < 0.05; ** *p* < 0.01). Comparisons were between the Intranasal and the Injection groups.

The anti-PRRSV serum IgG levels were evaluated using ELISA (S/P). Piglets in the control group were anti-PRRSV IgG-negative throughout the study. Moreover, the IgG levels in serum samples from inoculated pigs elevated gradually with post-inoculation until the end of the study (Figure 3A,B). The intranasal group had an earlier serum IgG response at 11 dpi within PRRSV-inoculated piglets. All the serum samples were antibody positive at 15 dpi. As shown in Figure 3C, the more robust serum IgG responses were detected before 27 dpi in the intranasal group. However, a stronger response appeared in the injection group after 31 dpi until the end of the current study.

### 3.4. Anti-PRRSV SIgA Detection in Saliva Samples 

**Figure 4:**
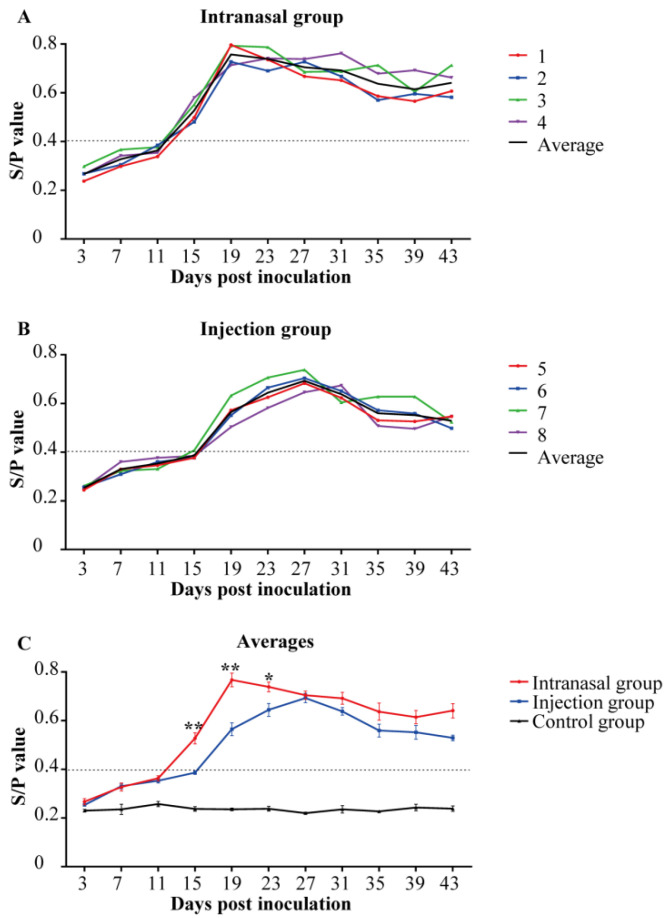
Anti-PRRSV saliva SIgA levels. The anti-PRRSV saliva SIgA levels were quantified using ELISA in piglets (*n* = 4 in each group) to assess the anti-PRRSV antibody response. Saliva samples collected at 0 dpi were served as the negative control. The cut-off for positivity was 0.4. Piglets in the control group were SIgA-negative throughout the study. (**A**,**B**): Colored lines represent the individual animals, and black lines represent the mean value of each group. Numbers (1–9) represent the individual pig numbers. (**A**) Anti-PRRSV saliva SIgA levels in the intranasal group. (**B**) Anti-PRRSV saliva SIgA levels in the injection group. (**C**) The averages of saliva SIgA levels within each group. The graph illustrates the mean ± SEM. Statistical differences were determined using the *t*-test (* *p* < 0.05; ** *p* < 0.01). Comparisons were between the Intranasal and the Injection groups.

The anti-PRRSV saliva SIgA levels were evaluated using ELISA (S/P). Piglets in the control group were anti-PRRSV SIgA-negative within the study. Within the PRRSV-inoculated piglets, anti-PRRSV SIgA became positive by 15 dpi and reached the highest levels at 19 dpi in the intranasal group. The SIgA levels stayed positive until the end of the study (Figure 4A). In the injection group, the positive result of SIgA levels began at 19 dpi, and the highest levels occurred at 27 dpi (Figure 4B). Moreover, from Figure 4C, positive SIgA was produced earlier and existed with higher levels in saliva samples from the intranasal group throughout the study. It indicated that intranasal inoculation had a faster and stronger saliva SIgA response than the injection inoculation.

### 3.5. Anti-PRRSV NAs Detection in Serum Samples

**Figure 5:**
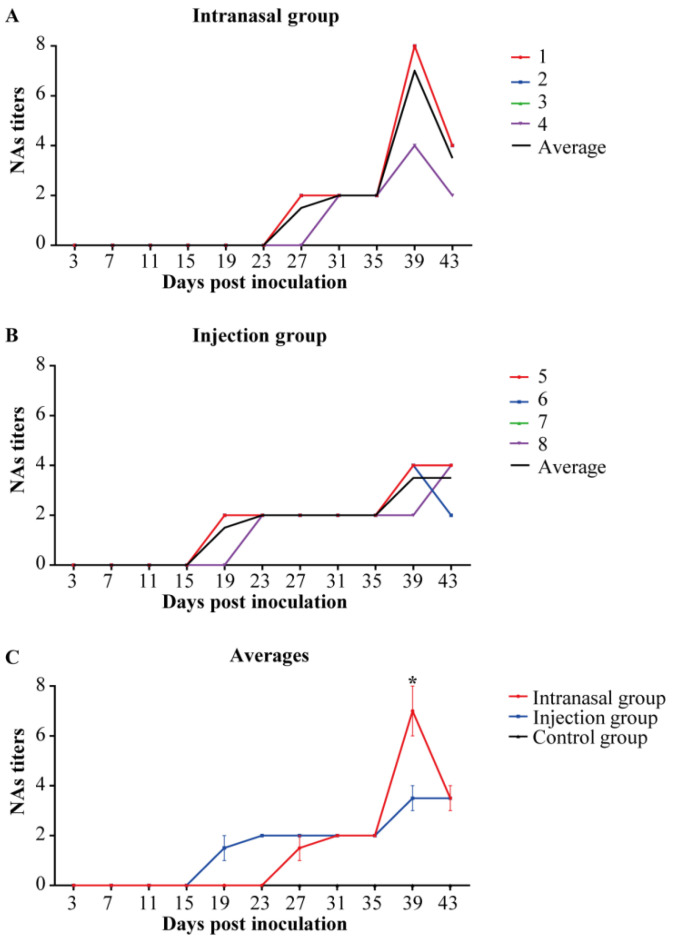
Anti-PRRSV serum NAs titers. The micro-neutralization assay results were determined by evaluating PRRSV RNA with RT-qPCR. NAs titers were below 1:2 in the control group in the study. (**A**,**B**): Colored lines represent the individual animals, and black lines represent the mean value of each group. Numbers (1–9) represent the individual pig numbers. (**A**) Anti-PRRSV serum NAs titers in the intranasal group. (**B**) Anti-PRRSV serum NAs titers in the injection group. (**C**) The averages of serum NAs titers within each group. The graph illustrates the mean ± SEM. Statistical differences were determined using the *t*-test (* *p* < 0.05). Comparisons were between the Intranasal and the Injection groups.

The anti-PRRSV NAs in serum samples are depicted in Figure 5. Figure 5A,B show that the serum NAs of low titers were produced at 19 and 27 dpi in the intranasal group (1:2–1:8) and injection group (1:2–1:4). The injection group produced NAs later in the intranasal group, but the NAs titers were slightly higher (Figure 5C). Thus, intranasal inoculation is more favorable for producing NAs than injection inoculation.

### 3.6. Anti-PRRSV NAs Detection in Saliva Samples

**Figure 6:**
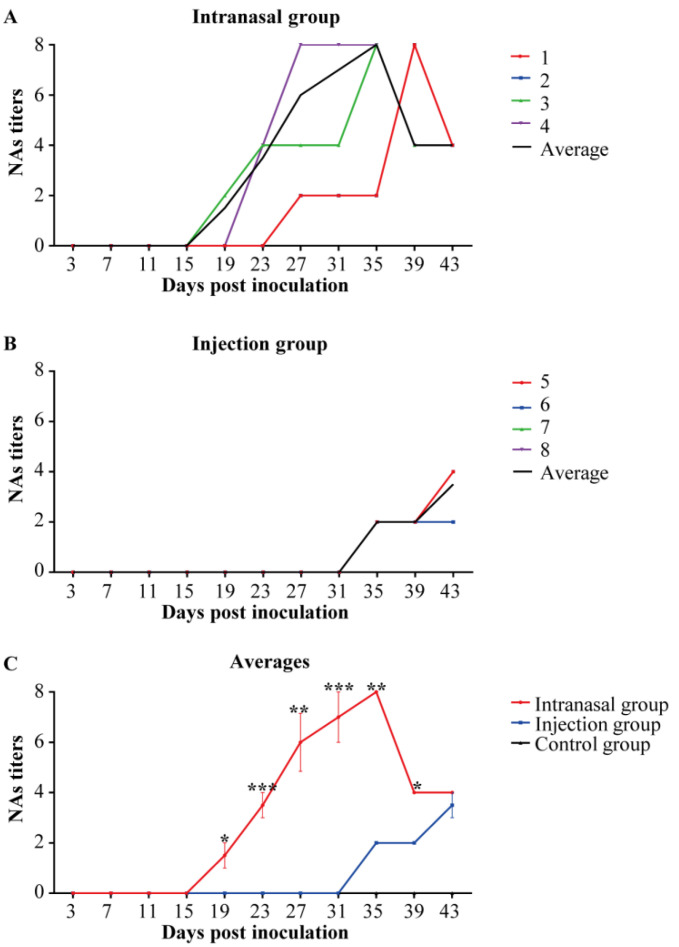
Anti-PRRSV saliva NAs titers. The micro-neutralization assay results were determined by evaluating PRRSV RNA using RT-qPCR. NAs titers were below 1:2 in the control group throughout the study. (**A**,**B**): Colored lines represent the individual animals, and black lines represent the mean value of each group. Numbers (1–9) represent the individual pig numbers. (**A**) Anti-PRRSV saliva NAs titers in the intranasal group. (**B**) Anti-PRRSV saliva NAs titers in injection group. (**C**) The averages of saliva NAs titers within each group. The graph illustrates the mean ± SEM. Statistical differences were determined using the *t*-test (* *p* < 0.05; ** *p* < 0.01; *** *p* < 0.001). Comparisons were between the Intranasal and the Injection groups.

The anti-PRRSV NAs in saliva samples are displayed in Figure 6. Figure 6A shows that the NAs of saliva (1:2–1:8) in the intranasal group were produced at 19 dpi, similar to the NAs in the serum. However, the NAs of saliva (1:2–1:4) in the injection group were produced at 35 dpi, later than the NAs in the serum (Figure 6B). As shown in Figure 6C, the intranasal group made previous NAs with higher titers than the injection group, indicating that the intranasal inoculation could induce an earlier NAs response.

## 4. Discussion

Since the porcine reproductive and respiratory syndrome outbreak was first reported in the late 1980s, it has affected the global swine industry and resulted in significant economic losses. Many strategies have been utilized to improve the efficacy and safety of PRRSV vaccines including developing different of vaccine types, various vaccinated procedures, and strict management. For over 30 years, vaccination was used to control PRRS as the primary strategy. However, it has achieved little success due to the inadequate understanding of PRRSV virology, origin, evolution, and the host immune response. The commercially available PRRSV attenuated vaccines have been considered more effective, but their efficacy and safety remain controversial [20]. 

Type I interferon (IFN-α and IFN-β) has crucial roles in the innate immune system defense against the viral infection by inducing an antiviral immune response [21]. Generally, PRRSV is a poor inducer of IFN-α. The PRRSV infection usually causes an inefficient or absent host immune response by inhibiting IFN-α production [13,14]. Similarly, MLV can reduced type I IFNs levels. Nevertheless, our previous studies have shown that the field virus HeN-3 induced a high level of IFN-α production in PAMs during early infection and slightly inhibited IFN-α production in PAMs during late infection [15]. Furthermore, HeN-3 can cause IFN-α production in piglets in the first 15 dpi [16]. Thus, HeN-3 infection can induce an effective IFN-α antiviral response. Accordingly, different PRRSV field isolates could differ in IFN-α induction. Therefore, it was speculated that the HeN-3 could be a potential candidate strain for MLV. Most previous studies have been primarily based on monitoring the immune responses inside the blood [22]. Recently, saliva samples have been increasingly used to surveil various pathogens by either antibody or nucleic acid-based assays [23,24]. Our results indicated that the virus and antibodies could be detected in serum and saliva after inoculation. By monitoring of the antibody-mediated immunity and virus shedding systematically and locally, we could manage to understand the dynamics of the humoral immune response post PRRSV inoculation.

The viral loads in collected samples from different groups were detected in the study. The results demonstrated that inoculations induced viremia within three days and lasted one month. Some animals depicted sporadic rebounds of viremia after clearance (Figure 1). This is in line with the current understanding of PRRSV viremia development [25]. The virus remained in the lung and lymph nodes after viremia disappeared, causing repeated viremia and viral persistence (Figure 2). However, the viral loads of the injection group were higher than the intranasal group. It has been reported that (1) PRRSV could significantly suppress the innate immune response and induce inflammatory injury through various mechanisms [26,27]. (2) PRRSV evades host cell-mediated immunity and causes the delayed onset of the Th1 immune response [28,29]. (3) PRRSV damages immune barriers [30]. In addition, PRRSV replicates in PAMs causes dysfunction or even cell death by necrosis and apoptosis [31], impairing their antigen-presenting function. Therefore, lower viral loads could reduce immunosuppression and decrease the contagiousness of the individuals. Therefore, reduced virus loads could be beneficial. The results depicted that the mucosal immune pathway could provide safer vaccination in using PRRSV attenuated vaccines because of the lower viral loads of PRRSV in the intranasal group. However, the virus can survive in some immune tissues after initial clearance in serum. Therefore, the PRRSV attenuated vaccines has a potential risk of spreading virus. 

The IgG and SIgA levels in the inoculated groups gradually increased after inoculation, and the trends of both groups were similar. By 1–2 weeks after the start of viremia, the IgG response started in all the groups. By 2–3 weeks after the onset of viremia, the SIgA response started in all the groups. Positive SIgA was detected earlier and presented with higher levels in saliva samples of the intranasal group throughout the study. Similar to the previous results, IgG antibodies were detected one week after inoculation, and maintained high levels at 2–6 weeks. The SIgA could be seen about two weeks after inoculation, appearing later than IgG [32]. In this study, viremia persisted in pigs despite positive antibodies. The PRRSV infection could induce antibody responses, but without any protection against PRRSV infection. Previous studies showed that PRRSV-ELISA antibody response did not well correlate with the protective immune status [33].

The NAs play a crucial part in the antibody response due to their blocking of viral infection [34,35]. Immunosuppression can limit and delay the occurrence of NAs. Therefore, improving the levels of NAs in vaccine research is essential. In addition, most neutralizing tests of PRRSV were based on Marc-145 cells (monkey kidney cells) but not the host target cell [33]. The primary target cell for PRRSV replication is PAMs. There are Fc receptors to the IgG antibodies on the surface of PAMs, and PRRSV-ADE infection enables the attachment and internalization of the virus onto the macrophages through Fc receptor-mediated endocytosis [36,37]. A recent study has found that high NA titer sera (1:96) tested on Marc-145 cells could not entirely block the PRRSV infectivity on its host target cell (PAMs) [38]. Therefore, neutralizing antibody testing in PAMs is more reliable. In this study, we established that anti-PRRSV antibodies had neutralizing ability in saliva and serum samples, and the PRRSV NAs response was coincided with the previous reports [8]. Similar to the serum NAs response, a low level of saliva NAs was developed at 3 or 4 weeks after inoculation, and the low NAs titers were between 1:2 and 1:8. Besides, NAs were produced earlier with higher neutralizing activity in the intranasal group. Therefore, the mucosal immune response of attenuated vaccines could elicit relatively adequate NAs during PRRSV infection. 

Mucosal vaccination pathways can elicit local and systemic immune responses to induce IgG and SIgA production. Nasal immunization has distinct features, protecting the site of pathogen invasion, and is more effective in preventing and treating infectious diseases, especially respiratory diseases, than traditional intramuscular vaccines. As a significant respiratory disease in piglets, there are only a few reports on mucosal immune responses of PRRSV. Intranasal vaccines are already available against influenza and others are under development [11]. CanSinoBIO utilizes atomized inhalation to train the immune memory function of the body by imitating the natural viral infection, effectively stimulating the triple protection of mucosal, humoral, and cellular immunity (https://www.nature.com/articles/d42473-022-00043-y, accessed on 18 April 2022). A range of novel nasal COVID-19 vaccines are being developed, and preclinical results have shown good prevention of replication and shedding of virus by inducing a mucosal immune response (SIgA) in the upper and lower respiratory tracts (URT and LRT), as well as robust systemic and humoral immune responses [39]. Moreover, intranasal immunization should be a mainly effective method to generate SIgA antibody responses in the upper and lower respiratory tract URT and LRT to neutralize and eliminate SARS-CoV-2 without inflammatory consequences [40]. These studies showed that PRRS vaccines elicit robust levels of NAs for efficacy protection. Thus, developing PRRSV mucosal vaccines can replace the traditional PRRSV vaccines given by the traditional immune pathways. It is vital that a PRRSV vaccine should protect pigs from the later PRRSV infection by inducing the production of high-level NAs, not only including NAs in the blood but also in the mucosae.

## 5. Conclusions

The results confirm that similar humoral immune responses have been induced in different inoculation modes. However, more robust antibody responses and lower viral loads could be generated during intranasal inoculation. Mucosal inoculation could be a better pathway for PRRSV vaccines, and future vaccine development needs to be based on strategies for increasing the level of neutralizing antibodies.
